# Compassion-focused group therapy improves depression, emotional eating, self-criticism and shame in people living with severe obesity: A single-centre, examiner-blind randomized controlled trial

**DOI:** 10.1371/journal.pone.0342744

**Published:** 2026-03-25

**Authors:** Mary Hynes, Francis M. Finucane, Chris Collins, Timothy O’Brien, Oliver McAnena, Grace O’Shea, Monika Pilch, Brian E. McGuire

**Affiliations:** 1 Bariatric Medicine Service, Centre for Diabetes, Endocrinology and Metabolism, Galway University Hospitals, Galway, Ireland; 2 Department of Medicine, College of Medicine, Nursing and Health Sciences, University of Galway, Galway, Ireland; 3 Department of Surgery, Galway University Hospitals, Galway, Ireland; 4 School of Psychology, University of Galway, Galway, Ireland; Tsukazaki Hospital, JAPAN

## Abstract

**Background:**

Severe obesity (defined as a BMI ≥ 40 kg m^-2^) is often accompanied by significant mental health co-morbidities such as eating disorders and depressive disorder and people living with severe obesity often experience feelings of shame, self-criticism and feelings of inferiority in relation to others. Compassion Focused Therapy (CFT) was specifically designed for people with high levels of shame and self-criticism and aims to promote self-compassion which is regarded as an adaptive emotional regulation strategy.

**Aims:**

To explore the effect on psychological outcomes of a 10-session (weekly for 2 hours) in-person, group-based CFT intervention for people living with severe obesity. We sought to determine whether CFT would lead to improved self-compassion, depressive symptoms, emotional eating, shame, self-criticism, submissive behavior, and negative social comparison.

**Method:**

A single-centre, randomized controlled trial was carried out with 91 participants allocated to either ‘treatment as usual’ (n = 46) or ‘treatment as usual with additional group based CFT’ (n = 45). Treatment as usual included dietary advice, assessment by a Consultant Endocrinologist with possible prescription of medication, and participation in an eight-week lifestyle modification programme. Psychological outcomes were recorded at three time points (pre-treatment, post-treatment, and three-months after the end of group-based CFT).

**Results:**

CFT led to statistically and clinically significant improvements in self-compassion, self-coldness, mood, shame, emotional eating, self-criticism, social comparison, and submissive behaviour (all *p* < 0.001) compared with treatment as usual. These results were maintained at three-month follow-up.

**Conclusions:**

Group CFT appears to be an effective psychological intervention to alleviate psychological distress in people living with severe obesity.

**Trial registration:**

ClinicalTrials.gov NCT03249441

## Introduction

People living with severe obesity (defined as a BMI ≥ 40 kg m^-2^) may experience high levels of depression and eating disorders [1], as well as shame, self-criticism and feelings of inferiority in relation to others [2]. Obesity-related stigma, defined as the social rejection and devaluation that accrues to those who do not comply with prevailing social norms of adequate body weight and shape [3], has been associated with increased difficulty losing weight and dietary and medication non-compliance [4], and can impact on shame, self-criticism and unfavourable social comparisons [5,6].

People living with obesity frequently experience a considerable degree of negative social judgment and terms such as “body shaming” have entered the common vernacular to describe this negative social judgment. Increasing research interest has focused on the relationship between shame and eating behaviour [7], for example, research shows that shame and self-criticism are particularly associated with binge eating symptomatology [8] and other forms of eating disorder [9].

A variety of individual and group psychological therapies have been used in obesity treatments, including cognitive-behavioral therapies (CBT) [10–12], acceptance-based therapy (ACT) [13], Mindfulness-Based Eating Awareness Training (MB-EAT) [14] and Compassion Focused Therapy [15]. Compassion-Focused Therapy (CFT) was specifically designed as a psychological intervention to address high levels of shame, self-criticism and self-directed hostility by activating affiliative and self-soothing systems [16]. When people with weight problems relapse or struggle to control their eating, they may become quite self-critical, even self-hating, which may increase difficulties with emotional coping and eating habits [17,18]. It has been suggested that even those in remission from an eating disorder experience higher levels of shame than other clinical groups [19] and disordered eating behaviour has been shown to increase following an experience of shame [20].

The literature on shame and obesity has demonstrated that individuals living with obesity are often subjected to both external and internalised stigma [21]. The presence of weight-related stigma exacerbates self-criticism, reinforcing a cycle in which feelings of shame and unworthiness lead to avoidance of health-promoting behavious and perpetuation of maladaptive coping mechanisms [22]. CFT helps self-critical and shame-prone individuals to cultivate an inner kindness and compassionate attitude towards their perceived personal shortcomings and distress [23]. Self-compassion is regarded as an adaptive emotional regulation strategy that helps create distance from suffering and transforms negative affect towards oneself into more positive, self-referential affection [24]. When individuals endure early experiences of abuse, criticism, or neglect or simply a lack of warmth and support, they may become more prone to self-criticism in times of distress [25]. According to Gilbert’s CFT model, it is only by developing a compassionate stance towards oneself and others that patients can break out of maladaptive coping cycles and take steps to recovery [25].

Self-criticism is significantly associated with shame-proneness, and both are transdiagnostic processes that are characteristic of many psychological difficulties, including depression, anxiety and disordered eating, increase vulnerability, affect expression of symptoms and elevate risk of relapse [26,27]. Blatt [28] conceptualised self-criticism as a core vulnerability factor for psychopathology, with individuals exhibiting high levels of self-critical perfectionism being particularly prone to psychological distress. Gilbert at al., further distinguished between different forms of self-criticism, highlighting the distinction between compassionate self-correction (which can be constructive) and self-hating self-criticism (which can be deeply destructive and often trauma-related) [29]. Self-hating self-criticism is of particular relevance to obesity, as individuals with early histories of trauma, neglect, or social rejection frequently internalise harsh, self-attacking narratives that perpetuate cycles of emotional eating, avoidance, and weight-related distress [30]. Kelly and colleagues have illustrated that those with an eating disorder demonstrated lower self-compassion and higher levels of ‘compassion fear’ than non-clinical participants [31].

Duarte et al. explored the understanding and experiences of 12 people living with overweight and obesity seeking help for weight problems. Using semi-structured interviews, they investigated the possible value and outcome of group-based, compassion-focused coping strategies for those seeking treatment for weight loss. This preliminary thematic analysis suggested that people living with overweight and obesity have problems with compassion, both receiving it from others and in being self-compassionate [32].

In terms of treatment effects, a meta-analysis of compassion-based interventions found that treatments not only produced significant moderate improvements in compassion (*d* = .55) but also significantly reduced symptoms of depression (*d* = .64), anxiety (*d* = .49) and psychological distress (*d* = .47) [33]. A systematic review concluded that CFT is a promising treatment for a range of mental health problems, especially when delivered in a group format over at least 12 hours [34]. Millard et al., in a meta-analysis, showed that CFT was more effective than control in improving self-compassion, self-criticism, depression and disordered eating in those with eating disorders, depression, PTSD/trauma-related symptoms, social anxiety, schizophrenia/psychosis, borderline personality disorder, and prolonged grief disorder [35]. Gouveia et al [36] and Hussain and colleagues [37] have linked higher self-compassion to greater psychological resilience, reduced emotional eating, and increased adherence to health behaviours. Forbes et al. demonstrated the efficacy of a CFT-based group program for women with obesity, finding significant improvements in self-compassion and internalized weight stigma, but this was an uncontrolled study [38].

To date, only one randomized controlled trial (RCT) [39] has examined CFT for psychological distress in people living with obesity. A 12-session, group CFT programme to reduce body-weight shame in 55 adults, showed that the primary outcome of shame (internal and external) was improved along with several other constructs including mood. This important RCT provided the first high quality evidence for the effectiveness of CFT for alleviating psychological distress in this population.

Following a successful pilot/feasibility study, we sought to further evaluate CFT in a randomized controlled trial to address self-compassion, depressive symptoms, emotional eating, shame, self-criticism, submissive behaviour, and negative social comparison in people living with severe obesity attending a specialist clinical facility for treatment of severe and complicated obesity.

## Methods

### Trial design

This study was a single-centre, randomized controlled trial with a 1:1 allocation ratio. The study design followed CONSORT guidelines.

### Trial registration and data availability

The trial was registered (ClinicalTrials.Gov trial registration number: NCT03249441 https://clinicaltrials.gov/ct2/show/NCT03249441) and the protocol document, based largely on the trial registration information, can be requested from the authors. Access to the data set is publicly available, interested parties can go to https://www.doi.org/ then search for the following https://doi.org/10.17605/OSF.IO/AUWKS to locate a copy of the dataset.

### Participants, eligibility criteria and settings

The study population consisted of patients referred for specialist psychological evaluation and intervention as part of the multi-disciplinary management of severe obesity in a regional (tertiary) hospital-based obesity service in Galway University Hospital, Ireland. The service caters to a mixed urban and rural catchment of around 750,000 people in the Western seaboard of Ireland. The recruitment period for the study commenced on 3/10/2016 and data collection was completed on 15/05/2017. The inclusion criteria were (1) severe obesity defined by a body mass index (BMI) of >40 kg/m² (2) over 18 years (3) no acute major psychiatric disorder (4) not in receipt of psychological intervention at the time of randomization (5) sufficient English language ability to take part in the group and complete questionnaires.

### Intervention and control conditions

Participants randomized to the control condition (Treatment as Usual group only (TAU)) attended the weight management clinic where typically dietary advice is given by a specialist dietician regarding weight management, there is medical and lifestyle advice from a clinical nurse specialist and assessment by a Consultant Endocrinologist with possible prescription of medication for management of diabetes and weight. All participated in an eight-week lifestyle modification programme [40]. Referral to the clinical psychologist can be made on a case-by-case basis by any member of the team when they consider that psychological or behavioural factors are central to the client’s weight difficulties.

Participants in the intervention group, ‘CFT +Treatment as Usual’ (CFT + TAU) arm received in-person, compassion-focused group therapy and were taught compassion-focused therapy exercises based on a manualized CFT group programme devised by Goss [41]. Group CFT occurred with a weekly session for 10 weeks, each session lasting two hours. See Table 1 for the content and session overview. All group sessions had the following format (1) review of the previous week’s independent practice with a focus on exploring the barriers and difficulties that patients faced (2) introduction of a specific CFT-related theme and practice of relevant CFT exercises within the group and (3) individual practice of compassion focused skills over the subsequent week. The CFT group sessions were delivered by a licensed Clinical Psychologist (MH), with advanced training in Compassion Focused Therapy. The CFT + TAU arm also attended the eight-week lifestyle modification programme. All TAU and CFT + TAU were conducted in person in the Diabetes and Endocrinology Department, Galway University Hospital.

**Table 1:** Description of the content and session overview of group Compassion-Focused Therapy.

**Week 1**	Understanding your relationship with food; What is meant by a Compasionate approach; Understanding the problem: Why we love to eat; The perils of modern society and supermarkets, Our need for Compassion; Moving away from moral judgements about eating.
**Week 2**	Making sense of overeating; How your body works; When survival skills backfire; Body issues; Health issues; Food, eating, and the need for self-compassion; What is a healthy body weight? How our bodies regulate their own weight: The Set-Point System; Overeating; Our patterns of eating; The Eating Response to Starvation, The Starve-Binge-Purge Cycle, Chaotic Eating, The Energy we use. Physical Activity. Excessive Exercise and Activity.
**Week 3**	Making Sense of Overeating. Eating your feelings; Learning to eat; Understanding our emotions; The CFT Emotional Regultion Systems. The Threat/Protection Systems: Emotions of Danger and Protection; The Drive System: Emotions of Achievement and Excitement; The Affiliative Soothing System: Emotions of Connection, Comfort, and Contentment; Soothing and Food. How are Food and Emotions linked? The Emotions affecting Dieting.The Social and Emotional Influences on Overeating. Overeating Rules and Self Criticism. Eating Rules in Action. Rules and Self Criticism
**Week 4**	The Compassionate Mind; What is Compassion and Why is it Important? Different States of Mind: Threatened Mind and Compassionate Mind. Food and our States of Mind; Food as Threat. Achievement Through Weight Loss. The Dieting Mind-Set.The Comfort-Food Mindset The Food as Fun Mindset. The Eat to Fit In, or Affiliative Eating Mindset. The Food as Punishment Mind-Set. Shifting Towards a Compassionate Mind; Our Compassionate Mind.: The Higher Picture. MultiModal Compassionate Mind Training. Compassion Attributes; Care for Wellbeing, Sensitivity, Sympathy, Distress Tolerance, Empathy. How it All Fits Together: The Skill of Self-Compassion.
**Week 5**	Preparing Your Mind for Compassion. Working with imagery. Choosing What We pay Attention To. Mindful Attention. Refocusing Our Attention. Activating your Affiliative Soothing System; Soothing Rhythmic Breathing. Developing your Safe Place; Imaging Your Safe Place and Feeling Safe. Feeling Wanted. Switching on The Compassionate Mind; Exercises to Develop The Compassionate Mind. Developing The Inner Compassionate Self. Wisdon, Strength, Warmth, Responsibility. Envisaging the Compassionate Self. You at Your Best. Compassion Every Day; Compassion Flowing Out to Others in Difficulty, Compassion for People Who Overeat; Compassion Flowing into You; Ceating a Compassionate Self-Compassion. Being the Focus of Self-Compassion. The Companion Other.
**Week 6**	Developing the Skill of Self-Compassion; Managing Distress Compassionately; The Vicious Cycle of Distress and Overeating. Learning to Activate your Affiliative Soothing System; Using Your Senses to Activate Your Affiliative Soothing System: Soothing by Sight, Soothing by Hearing, Soothing by Smell, Soothing by Touch, Strategies for Coping with a Crisis. Distraction Techniques; Deciding to Tolerate Distress; Learing to Tolerate Distress. Compassionate Thinking and Behaviour; Compassionate Thought Balancing. Getting Outside Your Mindset. Self-Critical Thinking versus Self-Compassionate Self Correction. Compassionate Behaviour. Keeping Diaries of Your Practice. Coping with Blocks to Compassion. Confusing Compassion with Pity.
**Week 7**	Why Do We Overeat: A Compassionate Approach; What A Compassionate Formulation For Overeating Involves. A Compassiomate Formulation for Overeating. Case Study. Key Threats. Current Triggers. Safety Strategy. Intended Consequences. Unintended Consequences. Pleasing others Comfort Eating. Eating as a Safety Strategy; Making Your Own Compassionate Formulation; Understanding your Current Eating Pattern; Talking to Your Compassionate Image. Writing A Compassionate Life Story. Understanding Your Current Eating pattern. Using an Eating Diary; Blocks to Monitoring; Managing Emotional Blocks. Using Your Diary to Make Sense of Your Eating.
**Week 8**	How Does Your Overeating work now? Overeating and the CFT Three Systems of Emotional Regulation; How Overeating May Help Us Manage Emotions. Case Study. Your Own Analysis of Eating and The Three Systems CFT Model. Putting It All Together: Creating A New Compassionate Formulation For Your Overeating.
**Week 9**	Motivating Yourself to Change; The Seven Stages of Change; Compassionate. Why Think About The Process of Change. Compassionate Motivation; Improving Your Chances of Success; Planning for Blocks to Change; Identifying Practical Problems. Overcoming Practical Problems. Getting Support to Help You Resolve Overeating; Identifying Sources of Help. Compassionately Managing Setbacks.
**Week 10**	Determining What Your Body Needs; Looking After Your Physical Needs. Appropriate Daily Calorie Needs and Balance of Food Types. Moving Towards Balanced Eating That Meets Your Needs.; Finding Your Energy Balance; Identifying Dificulties in Calorie Estimating. Compassionate Thoughts and Actions for Estimating Your Energy Intake. Figuring Out Your Energy Balance. Towards A New Way of Eating; The Six Step Program. The Beneftis of Meal Planning and Potential Problems; Learning to Respond to Feelings of Hunger and Fullness. Changing Food Associations. Caring for Your Body with Commpassion. Compassionate Letter Writing. Activate Your compassionate Image. Imagine a Compassionate Future. A Compassinate Focus on Eating.

### Outcomes (primary, secondary)

The primary outcomes for the trial were self-compassion and self-coldness (measured on the same scale), while secondary outcomes were social comparison (or self-worth), submissive behaviour, shame, self-criticism and the ability to self-reassure, mood and emotional eating.

### Sample size calculation

Feasibility of the study including recruitment and retention was carried out in a single-arm pilot phase of the study in which positive changes were observed in the outcome measures. Power analysis was conducted with the use of G* Power software for a one-tailed independent-samples t-test and indicated that the minimum sample size to yield a statistical power of at least.8 with an alpha of.05 and a medium effect size (*d* = 0.5) was 102 (51 per group).

### Randomization, random number generation, allocation concealment, implementation

Independent block randomization was conducted using a web-based computer-generated permuted procedure (http://www.random.org) in order to achieve a ratio of 1:1 to either CFT plus usual care or usual care only. Written informed consent was provided by each participant and witnessed by a research assistant who was not part of the research team.

### Blinding

Group allocation and outcome analysis were completed by an independent research assistant who was not part of the study.

#### Outcome time-points.

Outcome assessments were completed prior to randomization, at completion of the intervention phase and at three months follow-up. The outcomes were analysed by a research assistant who was blind to the participants’ group allocations and was not in any way involved in intervention delivery.

#### Interim analyses and stopping guidelines.

Not applicable.

### Materials

The outcome measures are described below.

#### Self-compassion.

The outcome of “self-compassion” was chosen because it is reflective of the main therapeutic target of CFT. Self-compassion was measured using the Self-Compassion Scale 12 item short form (SCS-12) [42]. Although there is some controversy about how the SCS factors should best be concepualised [43], a recent factor analysis of the SCS-SF [44] indicated that the most robust factor structure comprises two distinct sub-scales, one measuring a positive appraisal (the self-compassion sub-scale) and one measuring a negative appraisal (self-coldness sub-scale) and this bi-factorial model was also supported in another study [45]. The six-item subscale assessing self-compassion reflects participants’ tendencies to treat themselves kindly and compassionately at times of distress, using scores from 1 (almost never) to 5 (almost always) so that a higher score reflects greater self-compassion.

#### Self-coldness.

The outcome of “self-coldness” was also chosen as it reflects another key therapeutic target of CFT, which also aims to reduce self-critical attitudes and harsh self-judgement. Self-coldness was measured using the 6-item self-coldness subscale of the SCS-SF, assessing participants’ tendencies to respond to personal difficulties with self-directed emotional coldness, using scores from 1 (almost never) to 5 (almost always), so that a higher score reflects more self-coldness.

#### Social comparison.

“Self-worth” was measured using the Social Comparison Scale (SCS) [46], which was developed to measure self-perceptions of social rank and relative social standing. Higher scores indicate a greater tendency to consider oneself as socially attractive, of high rank and fitting in with others in society, while lower scores point to feelings of inferiority. Participants made a global comparison of themselves in relation to other people and rated themselves on a 10-point scale. The SCS has good reliability, with Cronbach’s alphas of 0.88–0.96 in clinical populations. The internal reliability for the present study was 0.89 using Cronbach’s alpha.

#### Submissive behaviour.

We measured “submissive behaviour” using the Submissive Behaviour Scale (SBS), which consists of 16 statements and respondents are asked to estimate the frequency with which they show these submissive behaviours [47]. The SBS presents a series of statements which describe how people act and feel in the context of social interactions and which in essence reflect self-esteem and self-worth in the context of social interactions, e.g., ‘I let others criticise me or put me down’, ‘I do what is expected of me, even when I don’t want to’, ‘I agree that I am wrong even though I know I am not’. Behaviours are scored on a five-point scale, with higher scores indicating greater feelings of submissiveness. The scale has good reliability, with a Cronbach’s alpha of 0.89, and 4-month test-retest reliability of 0.84 with a student population. The internal consistency in our study was 0.87 using Cronbach’s alpha.

#### Shame.

We quantified “external shame” using the Other as Shamer (OAS) scale [48] – this 18-item scale asks participants to respond to statements such as ‘I think that other people look down on me’ on a five-point Likert scale according to the frequency with which they make certain evaluations about how others judge them (0 = never to 5 = almost always). High scores indicate high external shame. The scale shows good reliability with a Cronbach’s alpha of 0.92 [49]. We found internal consistency of 0.92 in our study, using Cronbach’s alpha.

#### Self-Criticizing/Attacking and Self-Reassuring Scale.

The Forms of the Self-Criticizing/Attacking and Self-Reassuring Scale (FSCRS) [50] was used to measure self-criticism and the ability to self-reassure. It is a 22-item scale, which measures the different ways people think and feel about themselves when things go wrong for them. A confirmatory factor analysis showed that both in non-clinical and clinical samples, the three-factor model of FSCRS used here is a reliable measure for assessing two forms of self-criticism (inadequate self and self-hatred) and a form of self-reassurance. High FSCRS scores indicate high self-criticism and self-hatred [48]. We noted Cronbach alphas for inadequate self, self-hatred and self-reassurance in this study of 0.88, 0.79 and 0.81, respectively.

#### Mood.

The Beck Depression Inventory-II (BDI-II) is a widely used, 21-item self-report measure of depressive symptomatology [51]. The BDI-II specifies symptom severity from non-clinical to clinical ranges and has demonstrated sound reliability and validity. A total score of 0–13 is considered minimal, 14–19 is mild, 20–28 is moderate, and 29–63 is severe. The BDI-II has demonstrated good internal consistency, good construct, convergent, concurrent and divergent validity as well as good re-test reliability (r = 0.65). We noted a Cronbach’s alpha of 0.78 in this study.

#### Emotional eating.

The Emotional Eating Scale (EES) [52] measures the use of eating to cope with negative mood. Responders were asked to rate the strength of their urge to eat (in one of five categories ranging from ‘no urge to eat’ to ‘overwhelming urge to eat’) in relation to 25 different emotions. The emotions presented were divided into three sub-groups: anger/frustration, depression and anxiety. In our study, the total scale score had an adequate internal consistency with a Cronbach’s alpha of 0.82. The total score ranges from 0 to 100 and is categorised 0–25: a very healthy relationship with food, 26–46: moderate emotional eating, and 47–100: high emotional eating.

#### Statistical analysis plan.

A mixed ANOVA with the between-subjects variable of group (two levels: CFT + TAU vs TAU) and a within-subjects variable Time (three levels: Pre-intervention, Post-intervention and three-months follow-up) was conducted. Greenhouse-Geisser corrections were used whenever Mauchly’s test indicated a violation of sphericity. The questionnaire data were screened for missing values (there were minimal missing values) and we used an intention-to-treat analysis where missing values were managed by using the last observation carried forward (LOCF). Data were examined for normality of distribution using histograms and Kolmogorov-Smirnov tests and also examined for statistical outliers. Effect sizes were calculated based on pair-wise comparisons (T1 vs T2 and T1 vs T3 scores) using partial eta squared (η^2^) where 0.01 indicates a small effect, 0.06 indicates a medium effect and 0.14 indicates a large effect. As an indicator of clinically significant change, we described the proportions in each group that achieved at least 50% improvements in symptoms. We checked for any baseline differences in the variables between groups. The statistical package SPSS Version 26 was used for all data analysis.

## Results

### Participant flow

**Fig 1:**
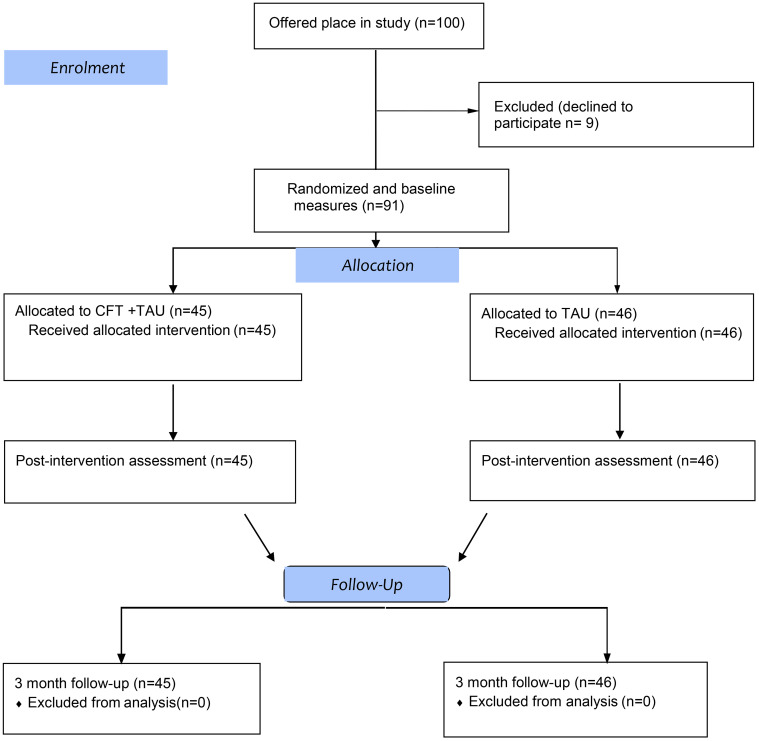
CONSORT flow diagram showing randomization, intervention and follow up in CFT + TAU and TAU groups.

Initially 100 participants were offered a place in the study but 9 dropped out before completion of baseline measures, leaving 91 participants in the trial, of whom 45 were randomized to CFT and 46 to usual care. All 91 participants attended all sessions and were retained at post-intervention and three-month follow-up and provided outcome data accordingly. The primary endpoint was after the 10-week CFT group intervention and the 3 month follow up was calculated from the end of treatment. Fig 1 shows the participant flow through the study.

The demographic and baseline clinical data for the sample are summarized in Table 2. The sample was classified as people living with severe obesity with a mean BMI in each group of 54 and the majority of the sample was female.

**Table 2:** Demographics and clinical characteristics at baseline with mean (SD) and p values.

Demographics and Clinical Characteristics at Baseline measures	CFT + TAU (n = 45)	SD	TAU (n = 46)	SD	*p*
Age (years)	47.6	7.35	48.8	10.58	
BMI	54	10.26	54.5	10.13	
Gender					
Male	9		7		
Female	36		39		
Self-Compassion Subscale	1.97	0.46	2.19	0.59	*p* = .052
Self-Coldness Subscale	3.70	0.52	3.65	0.50	*p = .597*
Social Comparison Scale	50.93	17.21	49.7	16.31	*p* = .739
Submissive Behaviour Scale	32.16	11.67	31.7	11.13	*p* = .778
Other As Shamer Scale	31.64	14.24	34.3	14.97	*p* = .388
Forms of Self-Attacking/Self-Criticism Scale					
(i) Inadequate Self	22.64	7.82	31.7	15.30	*p* < 0.05
(ii) Hated Self	10.91	8.53	10.7	7.77	*p* = .910
(iii) Reassuring Self	14.44	7.17	13.7	6.13	*p* = .593
Emotional Eating Scale	45.6	23.32	44.5	22.30	*p* < 0.01
Beck Depression Inventory – II	18.76	11.16	12.7	8.92	*p* = .819

The CFT and TAU groups differed significantly (*p* < 0.05) at baseline levels for measures of depression and inadequate self, with the CFT group scoring higher than the TAU on depression and the TAU scoring higher than the CFT group on inadequate self. The Beck Depression II scores were not at the clinical level for either group at baseline, indicating mild levels of depression.

### Clinical outcomes

In our exploration of the distribution of the outcomes, we noted that there were no statistical outliers for any outcome, but some extreme values were noted. Taking into consideration our review of the individual cases, our assessment is that these extreme values represent natural variations rather than errors or unusual data points.

Mixed ANOVAs with the between-subjects variable of group (two levels: CFT + TAU vs TAU) and a within-subjects variable Time (three levels: Pre-intervention, Post-intervention and 3 months follow-up) was conducted on scores of the Self-Compassion Scale, Social Comparison Scale, Other as Shamer Scale, Inadequate Self Subscale, Hated Self Subscale, Reassured Self Subscale, Submissive Behaviour Scale, Emotional Eating Scale, and the Beck Depression Scale-II.

**Self-Compassion Scale – Short Form (SCS-SF) Self-Compassion Subscale**. There were significant main effects for time, *F*(1.44, 127.78) = 245.04, *p* < .001, *η²p* = .73 and group, *F*(1, 89) = 36.15, *p* < .001, *η*²*p* = .29 and a significant Time x Group interaction, *F*(1.44, 127.78) = 149.77, *p* < .001, *η²p =* .63, indicating that the two treatment groups showed different patterns of change in self-compassion over time. Examination of means revealed distinct trajectories for each group. The TAU group showed minimal change across time points (T1: *M* = 2.19, *SD* = 0.59; T2: *M* = 2.38, *SD* = 0.54; T3: *M* = 2.36, *SD* = 0.55). In contrast, the TAU + CFT group demonstrated substantial improvements from baseline to post-treatment (T1: *M* = 1.97, *SD* = 0.46; T2: *M* = 3.38, *SD* = 0.58), with gains maintained at follow-up (T3: *M* = 3.43, *SD* = 0.53). The effect size for the Time x Group interaction was large (*η²p* = .63), indicating that 63% of the variance in self-compassion change patterns was explained by group allocation. By follow-up, participants in the TAU + CFT condition scored 1.5 points higher on the self-compassion scale compared to those receiving TAU alone, representing a clinically meaningful difference in self-compassion levels.

#### Self-Compassion Scale – Short Form (SCS-SF) Self-Coldness Subscale.

There were significant main effects for time *F*(1.20, 107.04) = 266.22, *p* < .001, *η²p* = .75 and group *F*(1, 89) = 33.33, *p* < .001, *η²p* = .27 and a significant Time x Group interaction *F*(1.20, 107.04) = 198.76, *p* < .001, *η²p* = .69, indicating that treatment groups showed different patterns of change in self-coldness over time. The pattern of change for self-coldness showed a complementary trajectory to self-compassion. The TAU group demonstrated minimal change across time points (T1: *M* = 3.65, *S*D = 0.50; T2: *M* = 3.55, *SD* = 0.49; T3: *M* = 3.59, *SD* = 0.50). Conversely, the TAU + CFT group showed substantial decreases in self-coldness from baseline to post-treatment (T1: *M* = 3.70, *SD* = 0.52; T2: *M* = 2.66, *SD* = 0.52), with reductions maintained at follow-up (T3: *M* = 2.66, *SD* = 0.52). The effect size for the Time x Group interaction was large (η²p = .69), indicating that 69% of the variance in self-coldness change patterns was explained by group allocation. By follow-up, participants in the TAU + CFT condition scored approximately one point lower on the self-coldness scale compared to those receiving TAU alone, representing a clinically meaningful reduction in self-coldness levels.

#### Social Comparison Scale (SCS).

There was a significant interaction effect for Intervention Type and Time, as shown in Table 3, *F*(2, 178) = 75.6, *p* < 0.001. Simple effects analysis indicated that participants’ Social Comparison scores increased significantly in the CFT + TAU group from Time 1 (*M* = 50.93) to Time 2 (*M* = 61.18), *p* < 0.001, η^2^ = 0.70 and remained improved at Time 3 (*M* = 60.29), *p* < 0.001, η^2^ = 0.70. Participation in the CFT + TAU group led to a significant improvement in Social Comparison scores while TAU was unchanged.

**Table 3:** Comparison of intervention and control groups on clinical outcomes on mean (SD) scores at post-treatment and 3-month follow-up, with effect sizes (η^2^).

Variable	Group	Baseline (T1)	Post-treatment (T2)	Effect size change from T1 to T2	3-month follow-up (T3)	Effect size change from T1 to T3
**Self-Compassion Subscale**	CFT	1.97 (0.46)	3.38 (0.58) a*	2.62	3.43 (0.53) b*	2.60
	TAU	2.19 (0.59)	2.38 (0.54) a	0.63	2.36 (0.55) b	0.43
**Self-Coldness Subscale**	CFT TAU	3.70 (0.52) 3.65 (0.50)	2.66 (0.52) a* 3.55 (0.49) a	2.47 0.53	2.66 (0.52) b* 3.59 (0.50) b	2.52 0.37
**Social Comparison Scale**	CFT	50.93 (17.2)	61.18 (15.4) a*	0.70	60.29 (15.3) b*	0.70
	TAU	49.76 (16.3)	50.13 (16.7)	0.02	49.05 (16.5)	0.03
**Submissive Behaviour Scale**	CFT	32.16 (11.7)	25.13 (11.3) a*	0.69	24.24 (10.9) b*	0.67
	TAU	31.67 (11.1)	30.11 (10)	0.25	30.02 (9.8)	0.18
**Other as Shamer Scale**	CFT	31.64 (14.2)	21.2 (11.3) a*	0.66	20.11 (10.6)b*	0.69
	TAU	34.3 (15)	32.83 (14.3)	0.26	32.63 (14.2)	0.26
**Forms of Self-Attacking/Self Criticism**						
**(i) Inadequate self**	CFT	22.64 (7.82)	15.02 (6.01) a*	0.70	14.84 (5.17) b*	0.69
	TAU	31.72 (15.3)	30.20 (14.12)	0.24	30.53 (14.51)	0.11
**(ii) Hated self**	CFT	10.91 (8.5)	7.16 (6.4) a*	0.41	6.82 (6) b*	0.42
	TAU	10.72 (7.8)	10.28 (7.5)	0.11	10.28 (7.6)	0.06
**(iii) Reassuring self**	CFT	14.44 (7.2)	20.09 (7.3) a*	0.70	20.07 (7.1) b*	0.71
	TAU	13.7 (6.1)	13.93 (5.9)	0.03	13.96 (5.9)	0.02
**Emotional Eating Scale**	CFT	45.6 (22.3)	27.96 (14.4) a*	0.53	26.11 (13.5) b*	0.56
	TAU	44.5 (22.3)	42.24 (22.1)	0.20	42.57 (21.7)	0.20
**Beck Depression Inventory**	CFT	18.76 (11.1)	13.87 (8.4) a*	0.60	12.76 (8) b*	0.56
	TAU	12.67 (8.9)	11.76 (8.1)	0.17	11.5 (7.9)	0.25

a = significant change from baseline to Time 2; b = significant change from baseline to follow-up.

* = significant difference between CFT and TAU, p value. < 001.

#### Submissive behaviour.

There was a significant interaction effect between Intervention and Time, *F*(2,178 = 38.76, *p* < 0.001. Simple effects analysis indicated that participants’ Submissive Behaviour scores reduced significantly in the CFT + TAU group from Time 1 (*M* = 32.16) to Time 2 (*M* = 25.13,), *p* < 0.001, η^2^ = 0.69 and to Time 3 *(M* = 24.24), *p* < 0.001, η^2^ = 0.67. The results indicate that participation in the CFT + TAU group led to a significant reduction in submissive behaviour which was maintained at Time 3 but TAU did not change.

#### Shame (Other As Shamer).

There was a significant interaction effect of Intervention Type x Time, *F*(2, 178) = 58.28, *p* < 0.001. Simple effects analysis indicated that participants’ Other as Shamer scores reduced significantly in the CFT + TAU group from Time 1 (*M* = 31.64) to Time 2 (*M* = 21.20), *p* < 0.001, η^2^ = 0.66, to Time (*M* = 20.11), *p* < 0.001, η^2^ = 0.69, while the TAU group showed no improvements.

#### Self-Criticism (Forms of Self-Attacking/Self-Criticism).

(i) Inadequate Self. Results indicated a significant interaction effect of Intervention Type and Time, *F*(2, 178) = 46.81, *p* < 0.001, whereby scores for the CFT + TAU group reduced from Time 1 (*M* = 22.64) to Time 2 (*M* = 15.02), *p* < 0.001, η^2^ = 0.70 and to Time 3 (*M* = 14.84), *p* < 0.001, η^2^ = 0.69. Overall, the results indicate that participation in the CFT + TAU group led to a significant reduction in Self-inadequacy score from Time 1 (pre-intervention) to Time 2 (post-intervention) and this effect was maintained at time 3, but no changes were seen in TAU. (ii) Hated Self: Results indicated a significant interaction effect of Intervention Type and Time, *F*(2,178) = 22.41, *p* < 0.001. Simple effects analysis indicated that participants’ Hated Self scores decreased significantly in the CFT + TAU group from Time 1 (*M =* 10.91) to Time 2 (*M* = 7.16), *p* < 0.001, η^2^ = 0.41 and again to Time 3 (*M* = 6.82), *p* < 0.001, η^2^ = 0.42, while the control group did not improve. (iii) Reassured Self. Analysis indicated a significant interaction effect between Intervention Type and Time, *F*(2, 178) = 73.24, *p* < 0.001. Simple effects analysis indicated that participants’ Reassured Self scores increased significantly in the CFT + TAU group from Time 1 (*M* = 14.44) to Time 2 (*M* = 20.09), *p* < 0.001, η^2^ = 0.70 and remained improved at Time 3 (*M* = 20.07), *p* < .0.001, η^2^ = 0.71 while TAU did not change.

#### Emotional Eating Scale.

Analysis indicated significant effect of Intervention Type and Time, *F*(2,178) = 39.54, *p* < 0.001. Simple effects analysis indicated that participants’ Emotional Eating scores reduced significantly in the CFT + TAU group from Time 1 (*M* = 45.60) to Time 2 (*M* = 27.96), *p* < 0.001, η^2^ = 0.53 and to Time 3 (*M* = 26.11), *p* < 0.001, η^2^ = 0.56. Overall, the above results indicate that only participation in the CFT + TAU group led to a significant reduction in Emotional Eating, while engaging in TAU did not lead to change.

#### Mood – Beck Depression Inventory II.

There was a significant interaction effect for Intervention Type and Time, *F*(2, 178) = 28.15, *p* < 0.001. Depression scores decreased significantly in the CFT + TAU group from Time 1 (*M* = 18.76) to Time 2 (*M* = 13.87), *p* < 0.001, η^2^ = 0.60, and Time 3 (*M* = 12.76), *p* < 0.001, η^2^ = 0.56, but TAU did not improve. As there was a baseline difference in the depression score, a sensitivity analysis was conducted to determine the robustness of our results. On the adjusted analysis, using an ANCOVA and using Depression Time 1 as a co-variate, it was found that nine effects remained stable and Hated-Self and Submissive Behaviour went from non-significant to significant for main effect of Intervention Type. The effect for change in depression symptoms remained significant.

The findings for all outcomes are summarized, with effect sizes, in Table 3.

The number of participants with scores on the primary and secondary outcomes improving by ≥50% at post-treatment and follow-up is shown in Table 4. Looking at the primary outcome of self-compassion, 78% improved by at least 50% at post-treatment, compared to only 4% for the TAU group. These effects in the treatment group were largely maintained at 3-month follow up (40% of participants achieved more than 50% improvement at follow-up). This pattern of greater numbers in the CFT + TAU group achieving at least 50% improvement was replicated across all variables (see Table 4). No adverse effects were seen in either group.

**Table 4:** Number (%) of participants with scores on the primary and secondary outcomes improving by at least 50% at post-treatment and follow-up in the treatment (n = 45) and usual care (n = 46) groups.

Variable	Group	Numbers having >50% change at post-treatment	Numbers having >50% change at follow-up
Self-Compassion Sub-Scale	CFT	35 (78%)	34 (76%)
	TAU	2 (4%)	2 (4%)
Self-Coldness Sub-Scale	CFT	1 (2%)	1 (2%)
	TAU	0 (0%)	0 (0%)
Submissive Behavior Scale	CFT	2 (4%)	3 (7%)
	TAU	0 (0%)	0 (0%)
Other as Shamer Scale	CFT	8 (18%)	7 (16%)
	TAU	0 (0%)	0 (0%)
Forms of Self-Attacking/Self Criticism Scale			
(i) Inadequate self	CFT	6 (13%)	7 (16%)
	TAU	0 (0%)	0 (0%)
(ii) Hated self	CFT	21 (47%)	23 (51%)
	TAU	0 (0%)	1 (2%)
(iii) Reassuring self	CFT	4 (9%)	6 (13%)
	TAU	0 (0%)	0 (0%)
Emotional Eating Scale	CFT	11 (24%)	17 (38%)
	TAU	1 (2%)	0 (0%)
Beck Depression Scale	CFT	9 (20%)	13 (29%)
	TAU	0 (0%)	0 (0%)

As the BDI-II utilizes a scoring range to indicate degree of severity of mood difficulty, we have reported the numbers in each category of severity (see Table 5).

**Table 5:** Numbers in TAU (n = 46) and CFT (n = 45) conditions in each level of depression category at baseline, post-treatment, and three-month follow-up.

BDI score	TAU Baseline	TAU Post-Treatment	TAU Follow-Up	CFT Baseline	CFT Post-Treatment	CFT Follow-Up
0-13 Minimal	29	30	30	17	21	26
14-19 Mild	6	6	6	6	9	8
20-28 Moderate	9	9	9	12	14	10
29-63 Severe	2	1	1	10	1	1

TAU = Treatment as Usual. CFT = Compassion Focused Therapy.

## Discussion

This study aimed to explore the effect on psychological outcomes of a 10-session (weekly for 2 hours) in-person, group-based Compassion Focused Therapy (CFT) intervention for people living with severe obesity. Specifically, we sought to determine whether CFT would lead to improved self-compassion, self-coldness, depressive symptoms, emotional eating, shame, self-criticism, submissive behaviour, and negative social comparison. Levels of self-compassion increased significantly in the CFT group and the effect was maintained at the three-month follow-up while levels of self-coldness reduced and this change was also maintained at follow-up. Of note, while the mean scores for those in the treatment group improved significantly on both the self-compassion and self-coldness sub-scales, 78% achieved a > 50% symptom change on the self-compassion sub-scale while only 2% of participants achieved >50% reduction in self-coldness. This might be interpreted as suggesting that the overall improvement was driven by enhanced self-compassion, which is, of course, the primary objective of the CFT model. Levels of self-criticism (hated-self and inadequate-self scores) reduced significantly following group intervention with large effect sizes and these changes were maintained at follow up. The results also confirmed high levels of shame and self-criticism in people living with severe obesity, consistent with previous research [31,38,39].

Although studies in the eating disorder literature have explored the role of self-criticism on outcomes, little is known about the impact of self-criticism in people living with severe obesity. All in all, self-criticism, self-hatred and self-reassurance appear to be important foci of therapy for people with severe obesity, and the CFT intervention had a promising impact on outcomes.

Consistent with other research findings, our study suggested that adults living with obesity share similar psychological characteristics with eating disorder populations, showing elevated levels of shame [8,20,32,38,39]. The expectation that practicing compassion would be associated with decreased levels of external shame, submissive behaviour and levels of social comparison were supported by the current findings, and improvements in these factors were maintained at 3-month follow-up.

Participants in the current study showed overall mild levels of depressive symptoms as measured by the BDI-II and similar to previous research [11,32], although levels of depressive symptoms were significantly higher at baseline for the CFT group. Of note, we observed significant reductions in the numbers with severe depressive symptoms in the CFT group.

Research has shown that emotional eating has been associated with worse weight outcomes, both in respect of weight gain over time and in difficulties with weight loss and weight maintenance [22,30]. Food is a powerful source of pleasure and reward, and previous research has shown that eating may serve the function of temporarily reducing negative affect and thus regulating complex emotions such as anger, anxiety, depression, shame and loneliness and trying to feel in control [32]. Our findings showed a significant reduction in levels of emotional eating in the intervention group but not in the control group. Indeed, levels of emotional eating were reduced further at 3 month follow up for the CFT group, with mean scores falling into the ‘normal eating’ classification. Some people living with severe obesity have learned that their eating behaviour can be a very useful way of managing affect. It is indicated in our research and previous research [32,38,39], that when people living with overweight or obesity relapse, or struggle to control their eating patterns, they can become self-critical, even self-hating which may increase difficulties with emotional coping and eating habits. Emotional eating can be an important variable for those people living with obesity as it has been found to mediate the relationship between over-eating and body mass change [32,33]. Thus, an intervention that can reduce emotional eating has many potential areas of application.

The research findings indicate that cultivating self-compassion and compassionate self-correction through CFT may offer improved emotional regulation strategies for people living with severe obesity. This psychotherapy model recommends that practitioners address the psychopathology arising from obesity as a main focus of therapy and to evaluate weight-loss *per se* as a secondary objective, although improved weight loss outcomes could well follow.

The only other randomized trial of CFT in this population [39] also found strong evidence of effectiveness, but the population was a little different – their population was based in primary care rather than a specialist weight management program and inclusion was based on BMI > 30 rather than >40 in our study. However, many of the findings showed similar levels of effectiveness despite the fact that our population might be considered more “severe”. Improvements of this magnitude appear to be clinically significant, especially without pharmacological intervention and these studies do seem to point towards an effective intervention to improve psychological health in a population of people living with obesity.

In terms of strengths of the study, this is just the second randomized controlled trial to explore whether CFT is associated with changes in psychological distress in a clinical population of people living with severe obesity. The high retention rate in this study was a strength, and this may be due to using an accessible, familiar clinic setting, where participants attended on a regular basis as was recommended from the pilot study. Maximising retention is important as increased attendance has been found to correlate significantly with weight loss in the longer term [41,53,54]. The use of an independent researcher, blind to the randomization process to administer the questionnaire and input the data reduced the risk of response bias. The study had a satisfactory sample size and the sample included both males and females.

The study had a number of limitations. The current findings are based only on the random samples derived from a single centre, and therefore future studies with much larger sample sizes from other geographical areas are needed to confirm the effectiveness of the intervention. Thus, these results cannot be generalized to all people living with severe obesity. There may be particular differences between individuals who seek medical treatment for weight difficulties and those who do not access treatment, for example, treatment-seekers may be more motivated to engage in some form of intervention. Another limitation is that all measures were based on self-report in this study. To overcome the shortcomings of self-report measures, researchers could employ objective physiological measures and interview-based rating scales could be developed as in previous research [22].

The use of Neff’s Self-Compassion Scale (SCS) has been criticised in the literature [8], in that the measure originates from a different theoretical tradition than Gilbert’s CFT model and there are ongoing debates regarding the measurement overlap between compassionate self-responding and self-criticism [43]. Our rationale for using the SCS was based on its widespread use, while recent studies psychometric properties have highlighted a valuable distinction between positive (self-compassion) and negative (self-coldness) ways of viewing oneself.

While the study was controlled in that both groups received usual care, the TAU group did not receive an equivalent amount of contact with a psychologist and a more closely matched control condition, such as an Acceptance Commitment Therapy or Mindfulness-Based Eating Awareness Training, could be considered for future studies. Nevertheless, the results have shown that CFT had a significant impact on emotional eating levels which have been shown in previous studies to be associated with weight difficulties. Although individuals recruited to the study were new to the Obesity clinic, they had varied weight management histories. This may have impacted on their engagement with the CFT or TAU and their approach to the study. Further rigorous randomized controlled trials with longitudinal data are needed to confirm the effects of CFT. Studies that examine the mechanisms of action, such as mediation and moderation studies, are also needed.

### Clinical implications

Overall, these findings suggest there may be an important role for psychological intervention to address psychological distress in severe obesity. Incorporating aspects of treatment that address levels of shame, self-criticism and psychological distress can effectively reduce levels of emotional and disordered eating, and improve mood, which have been shown to enhance the effectiveness of weight-management interventions [23,39]. If shame and self- criticism maintain eating disordered behaviour and are not addressed, weight loss interventions will not be successful and shame will be further reinforced due to the perceived failure of the individual to lose weight.

It is evident from the current research findings that targeting psychological factors such as shame, self-criticism and compassion through CFT, can significantly impact on the psychological wellbeing of people living with severe obesity, and this benefit was maintained at 3-month follow up assessment.

## Conclusions

We found that CFT led to a significant improvement in measures of self-compassion, self-reassurance, social comparison, shame, self-coldness, self-criticism, submissive behaviour, depressive symptoms and emotional eating in a group of individuals with severe obesity, as compared to the TAU control group. All therapeutic effects were maintained at 3-month follow-up. Thus, CFT may have an important role in treating the psychological distress associated with living with severe obesity. This study adds to the growing body of research supporting the application of CFT in obesity treatment.

## Supporting information

S1 File CONSORT 2010 checklist. (DOCX)

S2 File Protocol. (DOCX)
