# Supplementary material for: Compassion-focused group therapy improves depression, emotional eating, self-criticism and shame in people living with severe obesity: A single-centre, examiner-blind randomized controlled trial
Source: PLoS One. 2026 Mar 25;21(3):e0342744. doi: 10.1371/journal.pone.0342744 (PMC13016351; doi:10.1371/journal.pone.0342744)
Supplement: S2 File — (DOCX) [file pone.0342744.s002.docx]

**Compassion-Focused Therapy for People With Severe Obesity.**

**Protocol**

Study Overview

This study aims to explore the effectiveness of a group psychotherapy intervention using Compassion-Focused Therapy (CFT) in adults with severe obesity. In particular, it aims to evaluate the principle that CFT can be used to reduce levels of shame and self-criticism in adults with severe obesity. CFT was specifically designed for people with high levels of shame, self-criticism and self-directed hostility by helping people to cultivate affiliative emotions and compassion. The current research aims to explore changes in levels of self-criticism, shame, submissive behaviour, and self-comparison in a severely obese population who are awaiting bariatric surgery. Depression and levels of emotional eating are included as secondary outcomes for research.

Research questions:

The research was guided by the following research question and hypotheses:

Does CFT significantly improve levels of self-compassion and social comparison?

Hypotheses:

- 1. It was hypothesised that CFT would show significant improvements in comparison to TAU on self-compassion and social comparison variables and that these improvements would be maintained at 3 months follow-up.
  2. It was hypothesised that CFT would achieve significant reductions in shame, self-criticism, and submissive behaviour variables in comparison to TAU and that these improvements would be maintained at 3 months follow-up.
  3. It was hypothesised that CFT would show significant reductions in emotional eating and depression variables in comparison to TAU and that these reductions would be maintained at 3 months follow up.

Design: This study will use a prospective, randomised controlled design using quantitative methods to investigate the effectiveness of CFT, using various measures pre-and post-therapy and at 3-month follow-up.

Participants: 90 individuals with severe obesity will be randomly assigned to one of two treatment groups

Conditions: Compassion-Focused Therapy (CFT) plus treatment as usual or Treatment as Usual alone (TAU).

Experimental conditions:

Compassion-Focused Therapy (CFT) plus TAU participants will be taught the main compassion-focused exercises in a group setting as outlined in 'The Compassion-Mind Guide to Ending Overeating: Using Compassion-Focused Therapy to overcome Bingeing and Disordered Eating' manual (Goss, 2011) over a ten session period (weekly for 2 hours), offered over a 3 month period. Self-criticism and shame will be key foci across sessions. Participants in the CFT arm will also receive Treatment as Usual. Self-report measures will be administered prior to the commencement of the CFT group and TAU group, and during the final groups and 3 months follow up. To reduce the 'demand' effect, scales will be administered by an Assistant Psychologist who is not involved in delivering the therapy.

Treatment As Usual is based in the Diabetes, Endocrinology and Metabolism Clinic in Galway University Hospital. The Weight Management Service provides assessment by a multi-disciplinary team of endocrinologists, dieticians, nurse specialists and clinical psychologists. Dietary advice is given by a specialist dietician regarding weight management, assessment by the Consultant Endocrinologist with possible medication for management of diabetes and weight, and participation in a healthy lifestyle education program.

Inclusion Criteria:

- Participant has a clinical diagnosis of Severe Obesity, as defined by a Body Mass Index (BMI) of 40 kg/m² or more
- Participant is aged 18 years or older
- Participant is not in receipt of psychological interventions at the time of randomisation to group

Exclusion Criteria:

- Participant has insufficient English language ability to take part in the group and complete questionnaires

Allocation**:**Randomized

Interventional Model**:**Parallel Assignment

Interventional Model Description: This study utilised a randomised controlled trial (RCT) research design to investigate the efficacy of a group based CFT intervention programme. Participants were randomly allocated to one of the two groups, CFT plus Treatment as Usual (CFT) or Treatment As Usual (TAU), and psychological measures were administered at three time points (pre-intervention, post-intervention and follow-up assessment at three months follow up). The primary outcomes included self-compassion, shame and self-criticism. Emotional eating and depressive symptoms were assessed as secondary outcomes.

Masking: Single (Outcomes Assessor)

Masking Description**:**Clients are randomly assigned to one of two groups, Treatment as Usual (TAU) or Compassion-focused therapy plus Treatment as Usual (CFT). The outcomes assessor is blind to which of the two groups participants are assigned to.

Primary Outcome = Self Compassion based on The Self-Compassion Scale, Short Form (SCS-SF). This is a 12-item self-report measure, developed by Neff (2003).

Secondary outcomes:

Shame based on the Other as Shamer Scale (OAS; Goss, Gilbert, & Allan,1994).

Self-criticism based on The Forms of the Self-Criticising/Attacking and Self-Reassuring Scale (FSCRS; Gilbert, Clark, Hempel, Miles, & Irons, 2004).

Submissive behaviour based on Submissive Behaviour Scale (Allan & Gilbert, 1997).

Social comparison based on Social Comparison Scale (SCS; Allan & Gilbert, 1995).

Tertiary outcomes:

Mood based on Beck Depression Inventory-II (BDII; Beck, Steer, & Brown, 1996).

Emotional eating based on The Emotional Eating Scale (EES; Arnow, Kenardy & Agras, 1995).

Analysis Plan:

Power analysis was conducted with the use of G* Power software for a one-tailed independent-samples t-test and indicated that the minimum sample size to yield a statistical power of at least .8 with an alpha of .05 and a medium effect size (d = 0.5) was 102 (51 per group). The CFT+TAU vs TAU groups will be compared on mean scores on the specified outcome variables using a mixed ANOVA at pre-intervention, post-intervention and 3 months follow-up. Effect sizes will be calculated and the percentage change in symptoms for each group will be calculated and compared including the proportions in each group that achieve at least 50% improvement in symptoms.

References.

Allan, S., & Gilbert, P. (1995). Social Comparisons Scale: Psychometric properties and relationship to psychopathology. *Personality and Individual Difference, 19*, 293-299.

Allan, S., & Gilbert, P. (1997). Submissive behaviour and psychopathology. *British Journal of Clinical Psychology, 36*, 467-488.Beck, Steer, & Brown, 1996

Arnow B, Kenardy J, Agras WS. (1995). The Emotional Eating Scale: the development of a measure to assess coping with negative affect by eating. *International Journal of Eating Disorders*, *18*, 79-90.

Gilbert, P., Clarke, M., Hempel, S., Miles, J. N. V., & Irons, C. (2004). Criticizing and reassuring oneself: An exploration of forms, styles and reasons in female students. *British Journal of Clinical Psychology, 3*(1), 31-50.

Goss, K. (2011). *The Compassionate Mind Approach to Beating Overeating: Using Compassion Focused Therapy*. London, UK: Robinson

Goss, K., Gilbert, P., & Allan, S. (1994). An exploration of shame measures-I: 'The Other as Shamer Scale'. *Personality and Individual Differences, 17*, 713-717.

Neff, K. D. (2003). The development and validation of a scale to measure self-compassion. *Self and Identity, 2*, 223-225.
